# Assessment of Layer-By-Layer Modified Nanofiltration Membrane Stability in Phosphoric Acid

**DOI:** 10.3390/membranes10040061

**Published:** 2020-04-03

**Authors:** Kirsten Remmen, Barbara Müller, Joachim Köser, Matthias Wessling, Thomas Wintgens

**Affiliations:** 1 School of Life Sciences, Institute for Ecopreneurship, University of Applied Sciences and Arts Northwestern Switzerland, Hofackerstrasse 36, 4132 Muttenz, Switzerland; 2 School of Life Sciences, Institute for Bioanalytics, University of Applied Sciences and Arts Northwestern Switzerland, Hofackerstrasse 36, 4132 Muttenz, Switzerland; 3 Chemical Process Engineering, RWTH Aachen University, Forckenbeckstrasse 51, 52074 Aachen, Germany; 4 Institute of Environmental Engineering (ISA), RWTH Aachen University, Mies-van-der-Rohe-Str. 1, 52074 Aachen, Germany

**Keywords:** LbL modified membranes, acid stable membrane, phosphorus recovery

## Abstract

Nanofiltration (NF) can enable P recovery from waste streams via retaining multivalent impurities from spent pickling acid. However, with the currently available membranes, an economically feasible process is impossible. Layer-by-layer modified NF membranes are a promising solution for the recovery of P from acidic leachate. LbL membranes show a high level of versatility in terms of fine tuning for ion retention, which is necessary to achieve sufficient phosphorus yields. However, the stability of layer-by-layer modified membranes during phosphoric acid (H_3_PO_4_) filtration needs to be further investigated. In our study, we show that a polyethersulfone hollow fiber membrane modified with four or eight bi-layers was stable during immersing and filtering of a 15% H_3_PO_4_ solution. A sulfonated polyethersulfone (sPES)-based hollow fiber LbL membrane was only stable during filtration. Thus, we show the importance of applying real process conditions to evaluate membranes. Another important aspect is the influence of the high ionic strength of the feed solution on the membrane. We show that a high ionic strength led to a decrease in Mg retention, which could be increased to 85% by adjusting the process parameters.

## 1. Introduction

Acids such as sulphuric acid, hydrochloride acid, and phosphoric acid are used in pickling bathes to treat metal surfaces. During this process, the acid is enriched with metal elements, therefore after a certain time the acid is spent and needs to be renewed. Even though there are possible technologies allowing acid recycling, the most common disposal route for spent mixed pickling acid is still neutralization of the acid using lime. The dissolved metals are precipitated as their salts. Hence, no resource recovery, neither of the metals nor of the acid, takes place [1].

This is mainly due to several drawbacks of the available technologies (e.g., in terms of energy consumption, high emission levels, low recycling, high fresh water supply) [1,2,3]. It also needs to be pointed out that various pickling acids have varying recycling potential. Electro dialysis and diffusion dialysis show good results for the purification and recycling of HNO_3_/HF, HCl and H_2_SO_4_ mixtures [1,2]. However, these technologies are not very suitable for spent H_3_PO_4_ recovery. The high energy consumption, which occurs when separating concentrated H_3_PO_4_, leads to an economically and ecologically unfavorable process [4]. Nonetheless, P recycling is essential as it is considered a critical raw material by the European Union [5]. In addition, the production of phosphoric acid has a huge environmental impact. Each tonne of phosphoric acid produced makes fivefold the amount of hazardous waste [6,7]. During production, raw H_3_PO_4_ must be purified of impurities such as Fe, Mg, Al, V, Zn, Cd, and Ca. This is mainly carried out using solvent extraction, involving an extensive pre- and post-treatment. Solvent extraction processes have significant environmental impacts due to their resource intensity [1,2,3]. Hence, currently no satisfactory “green” recovery technology for H_3_PO_4_ from spent pickling acid is available.

Nanofiltration (NF) is a well-established process for all kinds of industrial separations, but spent pickling H_3_PO_4_ recovery is not one of them yet [3]. The application of NF for spent pickling acid and pickling baths, especially H_3_PO_4_, is still an emerging technology [3,8]. The applicability of NF was shown for P recovery from sewage sludge [9,10,11]. P concentrations in the acidified sewage sludge ash are much lower than in pickling acids. Nevertheless, the transport mechanisms of P through the membrane is the same. In the studies by Schütte et al. or Niewersch, commercially available membranes were used to retain metals and heavy metals but allow the permeation of P in the form of H_3_PO_4_. The concept was proven to be successful, yet the process is not economically feasible due to high P retention and low fluxes [9,10,11]. Schütte et al. concluded that for the recovery of metal contaminated H_3_PO_4_ solutions, a highly permeable membrane with a low retention for H_3_PO_4_ and highly positively charged ion retention is favourable.

Currently, such a membrane is commercially not available. This problem can be overcome by modifying membranes using polyelectrolyte (PE) layer-by-layer (LbL) deposition, creating NF membranes with tailor-made characteristics [12,13,14,15,16,17,18,19,20,21,22]. LbL membranes are fabricated by depositing oppositely charged PE on an also charged support material, in general a porous ultrafiltration (UF) membrane.

Due to the high fluxes, this novel technique was successfully applied, overcoming the above mentioned drawbacks (low flux, low P-yield) of commercially available membranes [12,23]. In addition, LbL assembly gives the membrane tailored properties such as charge, hydrophilicity, chemical resistance, high flux, resistance to fouling and therefore an overall improvement of the separation process.

However, little is known for such membranes with respect to acid resistance, acid permeability and impurity rejection. In the two previous studies carried out by us we successfully showed that LbL membranes outperform commercial membranes in terms of permeability and P recovery from an acidic environment [12,23]. It was shown that over 80% permeate recovery can be reached by filtrating with 10% phosphoric acid, while constantly achieving less than 20% P retention. Hence, it was possible to reach at least 75% P recovery. This recovery rate was achieved using a PES(PDAMAC/PSS)_6_ membrane [12]. However, these studies do not describe the impact of phosphoric acid on the stability of the membrane.

Besides our studies, very few publications have reported on the behavior of PE in an acidic environment. In most of these studies either weak PE were chosen, which were not stable at low pH levels, or the pH was not decreased below 3.5, as is needed to truly estimate the behavior of LbL membranes during harsh acidic applications [24,25,26], whereas strong PE such as PDAMAC and PSS are constantly charged independent of pH. Menne et al. [13] also showed excellent pH stability (0–14) of PE coated on ceramic UF membranes in HCl. In the described study, a ceramic membrane coated with three bi-layers was exposed up to a 1 M HCl solution for several hours and was shown to remain stable [13]. We also reported good stability for polymeric LbL membranes in a 1 M HCl solution [27]. In a study by Nehme et al. [28], coated capillaries were rinsed with 1 M HCl for 30 min. The coating of the capillaries was not affected by this acidic treatment [28]. These studies showed that the polyelectrolyte membranes (PEM) were stable in a harsh acidic environment [28,29,30].

These promising approaches were picked up on in this study and further developed to investigate its application to H_3_PO_4_ recovery in industrial processes. One parameter investigated in our study was the retention values for a highly osmotic solution. This is especially interesting in terms of later applications in acidic solutions, which often contain large amounts of salts and metals. Previous studies have shown that even though PDADMAC/PSS multilayer coatings are stable for a month if preserved in deionized water, they are highly sensitive to ionic strength, shear forces, and temperature changes [31,32,33]. Gao et al. pointed out that high ionic strength leads to variations in PDADMAC/PSS structure; however, in their case, the terminating layer was PDADMAC, which is known to be more sensitive to swelling than other PEs [31]. A change in the structure of the layers does not necessarily lead to a poor performance of the membrane. Hence, it is important to observe the properties of LbL membranes in highly osmotic solutions.

The stability of LbL-modified membranes in a harsh acidic environment was investigated to identify the potential of LbL membranes for use in acidic filtration tasks, taking H_3_PO_4_ as an example, due to its direct scarcity [34]. The change in performance of the LbL-modified membrane when exposed and during permeation of H_3_PO_4_ was investigated. This approach allows the prediction of potential applications in industrial processes and also highlights the need to further fine tune the membranes for acid separation applications.

## 2. Materials and Methods

Polyethersulfone (PES)- and sulfonated polyethersulfone (sPES)-based hollow fiber UF membranes with an inner diameter of 0.8 mm were provided by Pentair (Enschede, The Netherlands). The properties of the untreated membranes are shown in Table 1. Both membranes are negatively charged at a neutral pH [14]. Due to the sulfonate groups, the negative charge of the sPES membrane is significantly higher at a neutral pH. However, the ζ-potential of the sPES increases with decreasing pH. In contrast, the charge of the PES remains stable over a wide pH range (3–6.5).

### 2.1. Module Preparation and Membrane Coating Procedure

The membranes were potted in modules containing either one or ten fibers. Each hollow fiber had an inner diameter of 0.8 mm, and a length of 300 mm. This resulted in total membrane surfaces of 7.5 cm^2^ and 75 cm^2^ respectively. The single hollow fibre modules were built by gluing one hollow fiber membrane into a polyurethane tube with an inner diameter of 4 mm using polyurethane glue. The ten-fiber modules were built using a PVC tube with an inner diameter of 10 mm, using polyurethane glue.

### 2.2. Layer-by-Layer Coating

Before LbL assembly, the already-potted membranes were soaked in deionized water (1–3 µS/cm) for at least 24 h. Afterwards, the modules were rinsed with deionized water for at least one hour, applying a flow rate of 1 L/h/fibre. No pressure was applied. In the next step, the membranes were modified via LbL coating using PES.

Considering the negative charge of the membranes at a neutral pH, the first PE applied is a positively charged poly(diallyldimethylammonium chloride) (PDADMAC), with a molecular weight of 400–500 kDa in a 20% solution. This chemical, as well as all other chemicals, was purchased from Sigma-Aldrich (Buchs, Switzerland). The positive layer was followed by negatively charged polysodiumstyrenesulfonate (PSS), with a molecular weight of 1000 kDa, in a 25% solution. Both PEs were diluted to 1 g/L in 0.5 molar sodium chloride solution prepared from salt available at >99% purity.

**Figure 1:**
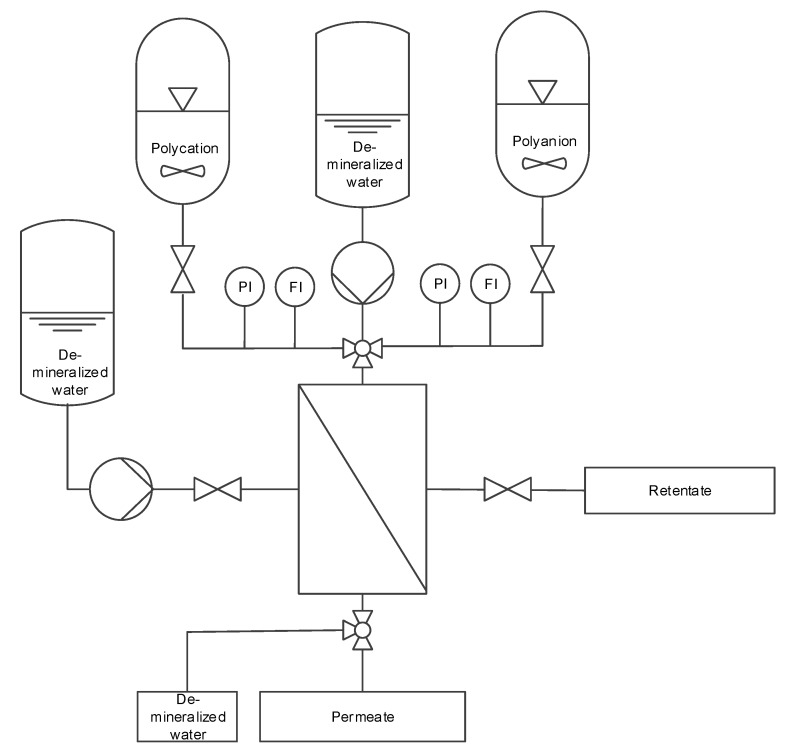
Flowchart of the coating process.

The PEs were applied using a dead-end filtration mode at a constant pressure of 3 bar (Figure 1). The coating unit consisted of three vessels with tubing for PDADMAC, PSS and demineralized water. The PE are deposited in an alternating manner on the membrane. During this step, the unit is run in dead-end mode. Between each deposition step, the membranes were rinsed with deionized water for at least 15 min and a flow rate of 1 L/h/fibre in cross flow mode. The conductivity of the rinsing water was measured in regular intervals using a GMH 3451 from Greisinger (Regenstauf, Germany). Rinsing was performed until a conductivity of below 3 µS/cm was reached and before the next coating step was carried out. The permeate side was also rinsed with demineralized water. The procedure was repeated until 4 or 8 bi-layers were deposited. A bi-layer consists of one positive and one negative PE layer.

Assuming that the PE is totally retained due to the pore size of the membrane, each applied layer can contain, depending on how many PEs are bound to the charged surface, a maximum of 2 g polyelectrolyte/m^2^ membrane area, resulting in a maximum of 4 g/m^2^ per bi-layer.

### 2.3. Single Hollow Fiber Modules Characterisation

Mg retention was used as an indicator of membrane separation performance. As a feed solution, 0.5 molar Mg_2_SO_4_ dissolved in deionized water was used. The solution was produced using Mg sulfate heptahydrate (>99% purity). H_3_PO_4_ (85 wt%) was diluted to 15 wt%, corresponding to a pH = 0.7. The pH was measured using a WTW inoLab Multi 9310 IDS pH-meter (Weilheim in Oberbayern, Germany). The following routine was carried out to evaluate the influence of phosphoric acid on the membrane.

1. Measurement of Mg retention in cross-flow mode at 5 bar of transmembrane pressure (TMP) using a 0.5 molar Mg solution. The flow volume was 80 mL/min, resulting in a cross-flow velocity of 2.65 m/s. This leads to a Reynolds number >2300, and thus, the experiments were carried out at turbulent flow conditions.

2. H_3_PO_4_ treatment (either (a) or (b) is applied)

(a) The lumen of the membrane is immersed in 15% phosphoric acid for 2 or 24 h.

(b) Cross-flow filtration at 5 bar was carried out using 15% phosphoric acid. During the entire treatment, phosphoric acid permeated through the membranes. The cross-flow velocity was 0.8 m/s. Due to the high viscosity of the phosphoric acid, this resulted in a non-turbulent but laminar flow. Again, this procedure was carried out for 2 and 24 h.

3. Repetition of step (a).

### 2.4. Multi Hollow Fiber Module Experiment

Membrane modules with ten capillaries were tested using a MaxiMem unit from PS Prozesstechnik (Basel, Switzerland). This unit allows the online measurement of the permeate flux during filtration. Again, the retention of MgSO_4_ was used as a reference to evaluate changes in membrane performance. The following routine was applied for the ten-capillary module experiment:

1. Measurement of Mg retention in cross-flow mode at 3, 5, and 7 bar TMP using 0.5 molar Mg-solution: The volume flow was 1000 mL/min, resulting in a cross-flow velocity of 3.32 m/s. This leads to a Reynolds number of approximately 3000. Thus, the experiments were carried out in a turbulent flow.

2. H_3_PO_4_ filtration for two hours: Cross-flow filtration at 3, 5 and 7 bar was carried out using 15% phosphoric acid. During the entire treatment, phosphoric acid permeated through the membranes. Again, the cross-flow velocity was 3.32 m/s.

3. The membrane was rinsed with deionized water until a neutral pH was reached.

4. Measurement of Mg retention in cross-flow mode at 3, 5, and 7 bar TMP using 0.5 molar Mg-solution

5. H_3_PO_4_ filtration as described in step (b), for another 22 h, hence in total 24 h of acidic filtration was applied.

6. The membrane was rinsed with deionized water until a neutral pH was reached.

7. Measurement of Mg retention in cross-flow mode at 3, 5, and 7 bar TMP using 0.5 molar Mg-solution.

### 2.5. Analytical Procedure

The Mg concentration of the samples was determined via triplicate analysis using ionic coupled plasma with optical emission spectroscopy (ICPOES) at a power of 1400 W (coolant flow: 13 L/min, auxiliary flow: 1 L/min, nebulizer flow: 0.75 L/min, Spectroblue SOP, Spectro Analytical Instruments, Kleve, Germany). The samples were diluted with 0.5 molar HNO_3_. Scanning electron microscopy (SEM) images were captured using a GeminiSEM from Zeiss (Jena, Germany). The membranes were broken in liquid nitrogen, dried, and sputter-coated with gold before imaging.

## 3. Results and Discussion

### 3.1. Evaluation of Membrane Permeability

Prior to any acidic treatment, the pure water permeability of all tested membranes was analyzed, which is summarized in Table 2. For all membranes, the permeability decreased by over 85%. The highest permeabilties were observed for the PES(PDADMAC/ PSS)_4_ membrane (17 L/(m^2^ h bar)), and the lowest for the sPES(PDADMAC/PSS)_8_ membrane. With a view on the initial flux of the bare membrane, the decrease was more evident for the PES membrane (98–99%) (Table 2). The decrease in permeability can be related to the closing of the pores (layer-dominated regime) or just decreasing the pore size (pore-dominated regime) [14]. The resistance of the membrane and hence also the permeability is influenced by this [13]. Yet, after coating, the difference in permeability for the four LbL membrane types is marginal. The permeabilities are similar to the ones reported by de Grooth et al. [14], for PES and sPES membranes. In other studies, higher permeabilities up to 40 L/(m^2^ h bar) were reported [13]. However, comparing LbL membrane performances is difficult, when the membranes are not coated exactly in the same way. Minor changes in the coating solution can have major influences on the membrane behavior. Nevertheless, this study will mainly focus on the influence of highly acidic feed solutions with a high ionic strength, as well as influence of the operating parameters.

### 3.2. Membrane Immersion in Phosphoric Acid Versus Filtration of Phosphoric Acid

**Figure 2:**
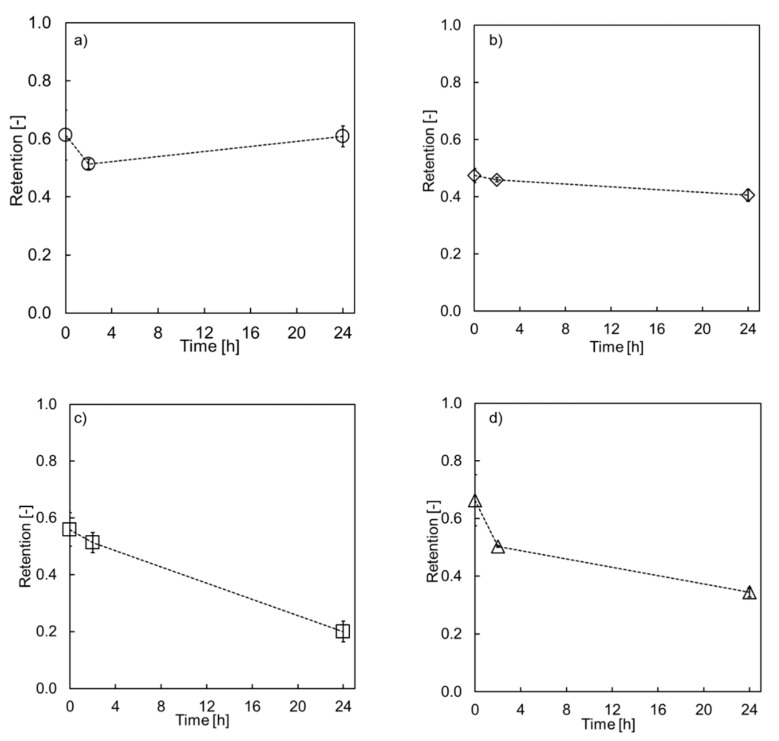
Mg retention values of immersed membranes in 15% H_3_PO_4_ (**a**) PES (PDADMAC/PSS)_4_, (**b**) PES (PDADMAC/PSS)_8_ (**c**) sPES (PDADMAC/PSS)_4_ (**d**) sPES (PDADMAC/PSS)_8_ as a function of immersion time.

During the first set of experiments, single fiber modules were tested. First, the lumen of the LbL modified membranes was immersed in 15% phosphoric acid. This concertation was chosen as it is close to real applications, e.g., during P recovery from sewage sludge ash. The results can be seen in Figure 2. As expected, the Mg retention before immersion was significantly increased for all modified membranes, in comparison to the unmodified membranes. MgSO_4_ retention can be found in Table 1 for the unmodified sPES and PES.

Each support membrane (PES and sPES) was coated with four or eight bi-layers of PE and each H_3_PO_4_ treatment was carried out in triplicate. For each experiment, a new module was used. Before immersing the lumen in 15% H_3_PO_4_ solution for 2 h and 24 h, Mg retention was measured. After 2 h and 24 h, each membrane was rinsed with deionized water until neutral pH was reached. The Mg retention was measured again and compared to the initial retention value.

The retention values for the membranes with the PES support layer were only marginally influenced by the acidic treatment. Over the time range of 24 h, the retention stayed almost constant. However, it has to be noted that for both layer variations (four and eight bi-layers), a slight decrease, of 2–10% in retention was observed after 2 h compared to the initial value. Several explanations can be found to describe this behavior: change in charge compensation, new alignment of the layers or decomposition of the layer [33,35,36]. In any case, it can be concluded that the influence of H_3_PO_4_ on both membranes (four and eight bi-layers) is negligible.

The membranes coated on a sPES support show a different picture. Again, a small change in retention was observed after 2 h of immersion, but a further decrease in retention was observed after 24 h. The retention decreased linearly with time for the sPES(PDADMAC/ PSS)_4_ membrane until only 20% of Mg retention remained after 24 h. The decrease for sPES(PDADMAC/ PSS)_8_ was less distinct than after 2 h, but still decreased down to 35% Mg retention from 65% initially. This might indicate that the number of layers, and therefore also the mass of PE on the membrane, plays a role in the chemical resistance towards an acidic environment.

The influence of the support membranes during backwashing cycles has already been shown by de Grooth et al. [14]. Here sPES membranes showed higher mechanical resistance towards the backwashing, which was ascribed to the higher charge of the sulfonated membrane. The layers assembled on the PES membrane altered during the backwashing cycle, resulting in decreased retention values [14]. During exposure to sodium hypochlorite, sPES membranes showed a great chemical tolerance towards the treatment [14]. No data on chemical resistance for PES nor sPES as a membrane support is known when exposed to low pH. Researchers have instead focused on the influence of PE on the support membrane properties, e.g., the mechanical strength [32], the ionic strength during coating [37] or the influence of PE pairing [38].

The results presented in this study indicate that the bond between sPES and the PDADAMC/PSS multilayer decreases when exposed to an acidic environment. However, immersing the lumen in H_3_PO_4_ is an extreme situation that usually only occurs during process interruptions. Hence, in the next step, filtration of H_3_PO_4_ was performed. Here, pressure was applied to the membrane as well as shear forces due to the cross-flow through the membrane.

**Figure 3:**
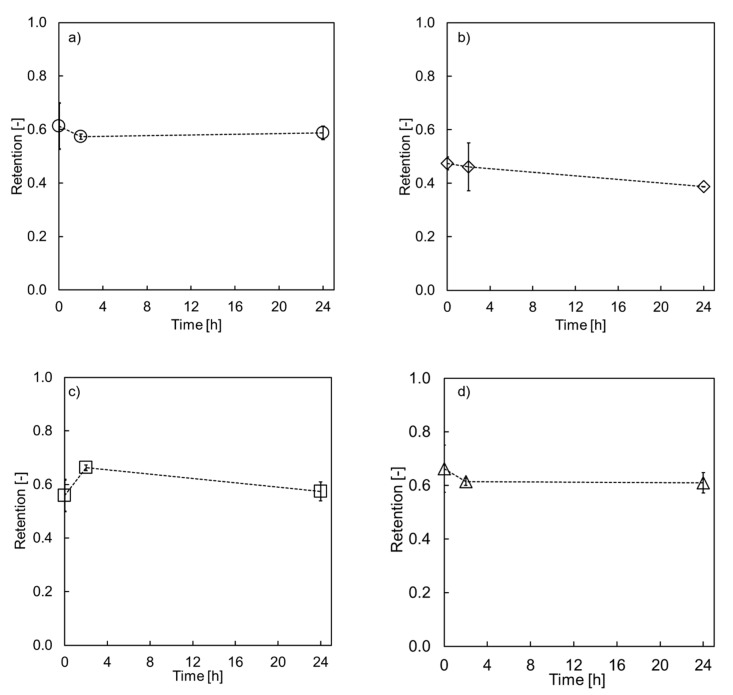
Mg retention values of membranes after filtering a 15% H_3_PO_4_ solution at TMP = 5 bar, v = 2.65 m/s (Re > 2′300) for (**a**) PES (PDADMAC/PSS)_4_, (**b**) PES (PDADMAC/PSS)_8_, (**c**) sPES (PDADMAC/PSS)_4_, (**d**) sPES (PDADMAC/PSS)_8_ as a function of H_3_PO_4_ filtration time.

A new set of membranes, equal to the ones tested in the first experimental phase, were prepared for the filtration of H_3_PO_4_. Instead of the former described immersion of the membranes in H_3_PO_4_, filtration was conducted at 5 bar for either 2 or 24 h. Mg retention was measured and used as an indicator for membrane integrity. The results shown in Figure 3 are very similar for all four membrane variations, showing almost no decreases in Mg retention. Neither the support layer nor the number of PE layers plays a role any longer. Again, slight changes after 2 h of filtration were observed, which can be ascribed to the membranes adjusting to the acidic conditions.

The applied pressure and cross-flow seemed to lead to a higher acidic resistance during filtration. The bonding of the PE to the substrate of sPES(PDADMAC/ PSS)_4_/sPES(PDADMAC/PSS)_8_ exposed to the acid may weaken during immersion as seen in Figure 2. The rinsing step after immersing washes the PE out of the lumen, and therefore subsequently measured Mg retention decreases. The longer the membrane is in contact with the acid, the more severe the damage resulting in decreased retention. After 24 h, similar retention values as for the bare membrane were reached. ζ-potential measurements showed that the charge for the sPES-based membrane becomes less negative with decreasing pH. This was shown for a pH range between 5.75–7.75 [14] and for a pH range between 2–6 [23]. At pH = 0.6, the membrane could even be less negatively charged as the sulfuric groups were no longer negatively charged. Hence, the former intrinsic charge compensation towards the support membrane was not stable anymore. Part of the layer will detach and wash out during rinsing with deionized water, leading to a non-dense and defective membrane. It seems that the stability of the LbL membrane is highly influenced by the pressure and cross-flow velocity during filtration.

**Figure 4:**
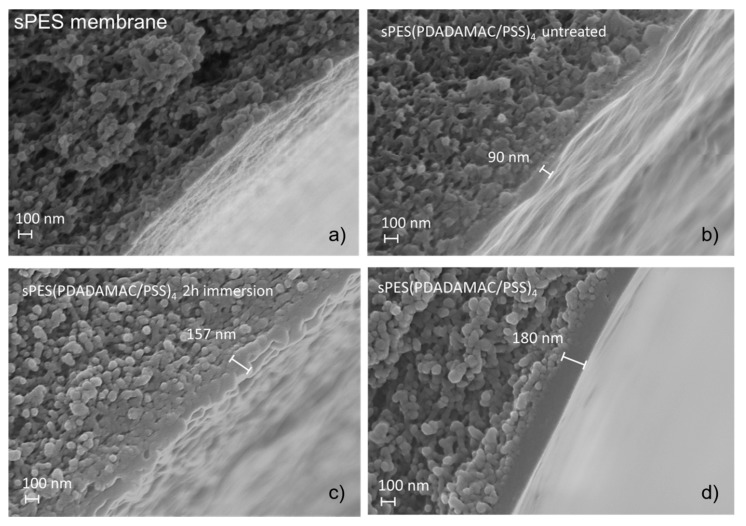
SEM images of sPES (sulfonated polyethersulfone) membranes; (**a**) uncoated membrane, (**b**) membrane after LbL coating, (**c**) coated LbL membrane after immersing for 2 h in phosphoric acid, (**d**) coated LbL membrane after 2 h of phosphoric acid filtration.

Other studies have shown that morphological changes caused by high ionic strength salt solutions can be reversed when rinsed with deionized water [33]. However, nothing has been reported on the filtration properties. Menne [30] showed a decrease in permeability after treating a PDADMAC/PSS system on a ceramic support membrane. The membranes tested in our study seem to alter irreversibly during the acidic treatment. This is supported by the SEM images in Figure 4, which are discussed in the next section.

### 3.3. Scanning Electron Microscopy Analysis of the Modified Membranes

The alteration of the LbL membrane was further examined by the example of sPES membranes using scanning electron microscopy. The performance of the sPES membrane changed depending if the membrane was just immersed in the acid without pressure and cross flow velocity or if filtration took place. SEM pictures were taken of (A) the bare sPES membrane; (B) the sPES(PDADMAC/ PSS)_4_ membrane directly after coating; (C) the sPES(PDADMAC/ PSS)_4_ membrane after immersing into H_3_PO_4_ for 2 h; (D) sPES(PDADMAC/ PSS)_4_ after filtering H_3_PO_4_ for 2 h. The image taken of the membrane after deposition of four bi-layers shows a membrane thickness of approximately 90 nm. This is in line with the results from Menne et al. [13], who obtained 140 nm for eight bi-layers and 70 nm for one bi-layer applying a dynamic coating.

Membranes coated with static coating have thinner bi-layers [36,39]. This indicates that in addition to the entropic forces mainly leading to the PE multilayer build up, additional binding regimes take place, leading to an increase in thickness during dynamic coating, as applied in this study. A deeper penetration into the membrane due to the applied pressure during coating could be a reason for thicker layers [13]. A potential explanation is partial entanglement of PE with the pores of the substrate membrane due to the applied mechanical forces. The “trapped” PE may not be washed out during the rinsing step with deionized water, allowing more PE to bind to the membrane, resulting in a thicker layer.

After immersing the membrane in H_3_PO_4_ for two hours, the layer was more swollen compared to the untreated sample (Figure 4C). The thickness of the immersed membrane was around 160 nm. The SEM picture indicates that the layer was no longer dense but rather porous like the UF substrate. The surface layer was rough and not homogeneous. This may be a consequence of the acidic treatment. The deteriorated morphology after immersing the membrane supports the data obtained from the experiments, showing lower retentions for Mg. Immersing the membrane in H_3_PO_4_ led to an altered membrane, which shows similar filtration characteristics as the bare UF membrane (Figure 2).

The image in Figure 4D shows the sPES(PDADMAC/ PSS)_4_ membrane after filtering H_3_PO_4_ for two hours. It can be seen that the PEM is thicker compared to the untreated layer (180 nm). However, the most significant features are the homogeneous structure and smooth surface of the layer. The surface is straightened and no defects can be observed. The influence of the cross-flow and the applied pressure most likely lead to this advanced surface of the membrane. Thus, it was shown that pressure and cross-flow velocity applied to the membrane are important factors to consider with respect to membrane alteration.

The SEM images support the hypothesis of severe damage to the PE multilayer during immersion and an intact layer during the filtration experiment. Pressure and cross-flow velocity applied during filtration seem to stabilize the membrane, and if a sPES membrane is used as a support, this is crucial to maintain an intact membrane (Figure 2 and Figure 3). Immersing the membrane into H_3_PO_4_ occurs usually only during an operational failure of the filtration process. Therefore, it can be concluded that in a regular working operation, the membrane performance is only slightly decreased for both support membrane types, after a steady state has been reached.

### 3.4. Influence of High Ionic Strength on the Mg Retention Using LbL Membranes

A study carried out by Bargeman [40] addresses the issue of membrane characterization only conducted for low salt concentrations, which does not reflect most of the industrial applications. In this study, retention values for a 500 mM MgSO_4_ feed solution are shown. The retention values range from 48 to 78%, which is rather low when compared to known data from the literature. Menne et al. [13] reached 80–90% MgSO_4_ retention for a sPES based support layer assembled with four bi-layered PDADMAC/PSS. De Grooth et al. [14] reached retention values of up to 84%, applying a PES based membrane with 2 bi-layers PDADMAC/PSS. However, these results were obtained using a 5 mM MgSO_4_ solution, whereas the results presented here show the retention for 500 mM MgSO_4_ solution, which is much closer to the salinity of industrial applications.

Several properties of the LbL led to decreased retention when filtering high osmotic salt solutions. Bargeman et al. [41] showed that as a consequence of an increase in ionic strength, the active layer swells, and hence, the retention values decrease due to increasing convective transport. Another important effect is the shielding of the membrane considering the high ionic strength of the feed solution. The counterions screen the membrane, and the repulsion effect for the coions is decreased [14,42,43].

**Figure 5:**
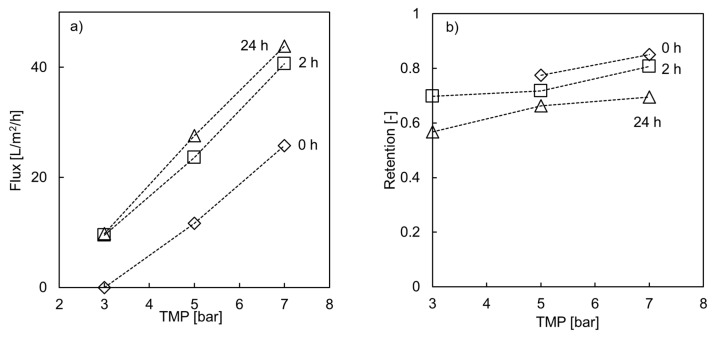
(**a**) Flux and (**b**) Mg retention for 500 mM MgSO_4_ feed solution for sPES (PDADMAC/PSS)_4_ as a function of TMP before and after filtering a 15% H_3_PO_4_ solution for two and 24 h (Re > 2’300).

In the next step, the possibility of increasing Mg retention values for LbL membranes by adjusting operating parameters is studied. In NF, retention values can be increased by increasing the convective water flow. This is achieved by increasing the TMP, thus increasing the overall transmembrane water flux. With increasing water flux, the share of mass transported by diffusion is decreased and hence plays only a minor role. Therefore, in the next step, a higher TMP was applied but also a higher cross-flow velocity influencing the membrane performance during acidic filtration. An increased cross-flow velocity can lead to an optimized transport regime at the membrane surface, e.g., by decreasing concentration polarization. The difference between the retention values of 5 mM and 500 mM MgSO_4_ was tested at a TMP of 5 bar. The results can be seen in Table 3 and Figure 5 for a PES(PDADMAC/PSS)_4_.

The retention values for a 5 mM MgSO_4_ feed solution were in the same range as the above-mentioned studies [13,14]. The retention values for a 500 mM solution could be increased to 77% at 5 bar, probably caused by the higher cross-flow velocity. By increasing the pressure to 7 bar, 85% Mg retention was reached. Due to a higher flux (Figure 5B) and therefore higher convective flows, the diffusive flow becomes smaller in relation, thus increasing the retention for Mg. It can be understood that even though many properties of an LbL membrane are defined by the chosen PE, coating conditions and feed solution, operational adjustment can also lead to an increased membrane performance. Phenomena typically influencing membrane performance such as concentration polarization and steric effects are decreased by optimized process parameters.

The influence of H_3_PO_4_ on flux and Mg retention as a function of TMP is shown in Figure 5 for a sPES(PDADMAC/ PSS)_4_ module with ten hollow fiber membranes. Through optimization of operating conditions, Mg retention was increased to 85% at 7 bar. It was not possible to observe a permeate flow at 3 bar at the beginning of the experiment (0 h), and hence also no Mg retention could be measured. This is due to the osmotic pressure of the bulk solution. According to the Van ’t Hoff equation for osmotic pressure Π (Equation 1) [44] and taking the water hold up of the membrane unit into account, the osmotic pressure for the feed solution is between 8.5–12 bar. Which is in line with literature values calculated by OLI Stream Analyzer [45]. Assuming a Mg retention of 70%, a pressure difference between 5.9–8.5 bar needs to be overcome. At a TMP of 5 bar, retention and flux values were able to be recorded at the beginning of the experimental time (0 h).

(1) $$\Pi =-\left(\frac{n_{s}}{V}\right)RT$$

where Π osmotic pressure, n number of moles of species, *V* volume of solvent, *R* gas constant, *T* temperature.

After two hours of acidic filtration, the retention values decreased slightly compared to the initial measurement. At 7 bar, a Mg retention value of 80% was recorded. After two hours of acidic filtration, a retention value of 70% at 3 bar could be recorded. This indicates that for LbL-modified membranes, the membrane resistance plays an important role [30]. The ideal permeate flux *Vp* through an uncoated UF membrane is described by the pore-model (Equation (2)) [46]. After modifications, the membrane shows NF properties; therefore, the transport might be better described by the extended NernstPlanck equation [46,47]. However, all of these models do not take into account the special properties of an LbL system. An increased water permeability can be ascribed to the swelling of the PDADMAC in the membrane [30,48]. Another influencing factor is the ion-exchange properties of an LbL membrane [37,48]. The osmotic strength of the PE multilayer itself also influences the properties of the membrane [30]. The osmotic strength can be influenced by the NaCl concentration of the coating solution. For LbL membranes, two forms of bindings can occur, which can be influenced by the ionic strength of the coating solution, and intrinsic and extrinsic charge compensation. Rather dense and thin layers are present if intrinsic charge compensation is the dominating regime, which results in high ion rejection of the membrane. A membrane with mostly extrinsic bound PE has a more open and swollen structure resulting in lower ion rejection [35,36]. Another influencing factor is the ion-exchange properties of an LbL membrane, which is not further discussed in this paper [37,48].

(2) $$V_{p}=\frac{\dot{m_{p}^{\prime \prime }}}{\rho_{p}}=A\Delta p$$

(3) $$A=\frac{\varepsilon^{3}}{\eta {\left(1-\varepsilon \right)}^{2}S_{\left(V\right)}^{2}2\tau \delta }\;(\mathrm{Carman}-\mathrm{Kozeny})$$

where $V_{p}$ permeate flow, $\dot{m_{p}^{\prime \prime }}$ permeate mass flow, $\rho_{p}$ permeate density, p pressure, ε membrane porosity, η dynamic viscosity, $S_{\left(V\right)}^{}$ volume specific surface area, τ tortuosity, δ membrane thickness.

Before H_3_PO_4_ filtration (at 0 h) the pure water flux increases linearly up to 25 L/(m^2^ h) at 7 bar. After 2 h of H_3_PO_4_ permeation the pure water flux for a sPES(PDADMAC/ PSS)_4_ membrane increased to 40 L/(m^2^ h) at 7 bar and after 24 h to 44 L/(m^2^ h) (Figure 5), compared to 25 L/(m^2^ h) as the initial value at 7 bar. Thus, the membrane is very likely to have altered due to the ion-exchange process during filtration, which leads to increased flux and decreased retention [37,48]. The rate of change in flux after 2 h is higher than between 2 and 24 h. This indicates that a steady state is reached.

## 4. Conclusions

To conclude, layer-by-layer modified membranes are applicable in a harsh acidic environment. This was shown for two different support layers: PES and sPES. The membranes were tested in two types of configurations during exposure to acidic conditions: Mg retention decreases slightly when applying pressure and cross-flow, but a steady state can be reached. However, if no pressure is applied, as during immersion, not all support layer materials are suitable. For sPES-based membranes, Mg retention decreases by over 40% during immersion. In contrast, Mg retention decreased only slightly if the layer was coated onto the PES support. To guarantee a process with an intact membrane and constant retention values, process interruption, in which the membrane is soaked in H_3_PO_4_ without pressure and cross flow, must be avoided.

Retention values for high-ionic-strength solutions are not as high as for solutions with a low ionic strength. However, by adjusting process parameters, such as cross-flow velocity and TMP, Mg retention values of up to 85% can be reached even for a 500 mM MgSO_4_ solution.

The main finding of this study is the possibility of tailoring LbL membranes to achieve an efficient and environmentally friendly solution for H_3_PO_4_ recovery and acid recovery in general. The next steps will be to apply LbL membranes to applications such as spent H_3_PO_4_ recovery or P recovery from sewage sludge.

## Figures and Tables

**Table 1:** Properties of virgin membranes [14].

	Permeability (L/m^2^/h/bar)	MgSO_4_ Retention (%)	MWCO (kDa)	ζ-potential (mV)
PES	1′100	-	100	−13
sPES	80	22	10	−19

**Table 2:** Permeability of coated membranes.

	Permeability (L/m^2^/h/bar)	Decrease Compared to Virgin Membrane (%)
PES uncoated	1′100	-
sPES uncoated	80	-
PES(PDADMAC/PSS)_4_	17	98
PES(PDADMAC/PSS)_8_	15	99
sPES(PDADMAC/PSS)_4_	10	87
sPES(PDADMAC/PSS)_8_	7	91

**Table 3:** Permeability of coated membranes.

MgSO_4_ Feed Concentration (mM)	Retention (-)
5	0.90
500	0.77
